# Orthopoxvirus Seroprevalence and Infection Susceptibility in France, Bolivia, Laos, and Mali

**DOI:** 10.3201/eid2812.221136

**Published:** 2022-12

**Authors:** Léa Luciani, Nathanaël Lapidus, Abdennour Amroun, Alessandra Falchi, Chanthala Souksakhone, Mayfong Mayxay, Audrey Dubot-Pérès, Paola Mariela Saba Villarroel, Issa Diarra, Ousmane Koita, Pierre Gallian, Xavier de Lamballerie

**Affiliations:** Aix-Marseille Université-IRD 190-Inserm 1207, Marseille, France (L. Luciani, A. Amroun, A. Dubot-Pérès, P.M. Saba Villarroel, I. Diarra, P. Gallian, X. de Lamballerie);; Sorbonne Université, Inserm, Saint-Antoine Hospital, Paris, France (N. Lapidus);; Université de Corse Pascal Paoli, Corte, France (A. Falchi); Lao Red Cross, Vientiane, Laos (C. Souksakhone);; Ministry of Health, Vientiane (M. Mayxay); University of Health Sciences, Vientiane (M. Mayxay);; Mahosot Hospital, Vientiane (M. Mayxay, A. Dubot Pérès);; University of Oxford, Oxford, UK (M. Mayxay, A. Dubot Pérès);; University of Sciences, Bamako, Mali (I. Diarra, O. Koita);; Établissement Français du Sang, La Plaine Saint Denis, France (P. Gallian)

**Keywords:** Orthopoxvirus, vaccinia virus, cowpox virus, monkeypox virus, seroprevalence, sexually transmitted infections, viruses, zoonoses, France, Bolivia, Laos, Mali

## Abstract

To determine a demographic overview of orthopoxvirus seroprevalence, we tested blood samples collected during 2003–2019 from France (n = 4,876), Bolivia (n = 601), Laos (n = 657), and Mali (n = 255) for neutralizing antibodies against vaccinia virus. In addition, we tested 4,448 of the 4,876 samples from France for neutralizing antibodies against cowpox virus. We confirmed extensive cross-immunity between the 2 viruses. Seroprevalence of antibodies was <1% in Bolivia, <5% in Laos, and 17.25% in Mali. In France, we found low prevalence of neutralizing antibodies in persons who were unvaccinated and vaccinated for smallpox, suggesting immunosenescence occurred in vaccinated persons, and smallpox vaccination compliance declined before the end of compulsory vaccination. Our results suggest that populations in Europe, Africa, Asia, and South America are susceptible to orthopoxvirus infections, which might have precipitated the emergence of orthopoxvirus infections such as the 2022 spread of monkeypox in Europe.

Immunity of human populations against viruses of the genus *Orthopoxvirus*, to which monkeypox virus (MPXV), variola virus, and vaccinia virus belong, has been questioned recently because of the emergence of MPXV infections. Broad cross-immunity exists between the viruses of this genus, which enabled the use of vaccinia virus as a vaccine to prevent smallpox. In addition, vaccinia virus–derived vaccines have been used to prevent or mitigate MPXV infections during the 2022 outbreak.

Since smallpox vaccination ended in 1980, immunity against orthopoxviruses has decreased worldwide. Decreased immunity has been associated with the emergence of zoonotic orthopoxviruses with extended host specificity. MPXV has been responsible for widespread epidemic episodes in Africa (*1*–*3*) and other continents (*4*,*5*). Similar episodes have been observed for buffalopox (*6*–*8*) and camelpox (*8*–*10*) viruses in Asia and for cowpox virus, which is ubiquitous (*11*–*13*). Orthopoxvirus infections will likely become more common because of increased travel and trade, ecosystem changes, and altered biodiversity and climates (*14*–*16*). Since smallpox eradication in 1980, medical research on orthopoxviruses has gradually declined. However, in 2001, several reports addressed the potential bioterrorism risk associated with smallpox (*17*–*19*). These reports led to attempts to assess the susceptibility of the general population to smallpox (*18*), which has generally been determined according to smallpox vaccination coverage. In 2001, the Santé Publique France (French Institute of Public Health) published a report using data from the country’s National Institute of Statistics and Economic Studies and National Institute of Health and Medical Research that estimated smallpox vaccination coverage in France (*20*). Coverage was ≈0% for persons born after 1979, 50% for those born during 1972–1978, 65% for those born during 1966–1971, and 90% for those born before 1966. 

The strategy to prevent smallpox in France and most developed countries was through systematic and mandatory vaccination of children. Vaccination consisted of 2 injections; the first injection was administered at 1 year of age and the second 10 years later. Smallpox vaccination in France was mandatory during 1902–1978 for the first injection and until 1984 for the booster. However, for many resource-limited countries, routine vaccination of the population was difficult to achieve, and the World Health Organization shifted to a containment strategy of case identification, isolation, and widespread vaccination of contacts in the 1960s. This strategy was successful in eradicating smallpox (*21*), but vaccination coverage of the general population in those countries (which conferred cross-immunity to other orthopoxviruses) was lower than in countries where routine vaccination had been organized.

We conducted a large-scale epidemiologic study of the prevalence of neutralizing antibodies against vaccinia and cowpox viruses. We tested ≈6,500 serum samples from persons in 4 countries on different continents: France, Bolivia, Laos, and Mali. We provide a demographic overview of orthopoxvirus seroprevalence that enables assessment of susceptibility of relevant populations to infection by this group of viruses.

## Materials and Methods

### Study Populations and Ethics Approval

We investigated human populations from France, Bolivia, Laos and Mali. We tested blood samples from all study participants for the presence of neutralizing antibodies against vaccinia virus. In addition, we tested a large cohort of the study participants in France for the presence of neutralizing antibodies against cowpox virus.

The population in France comprised 4,876 voluntary, unpaid blood donors whose serum samples were collected in 2012, 2013, and 2019 from 4 regions of metropolitan France: Auvergne-Loire (n = 837), Corsica (n = 596), Midi-Pyrénées (n = 1,738), and Provence-Alpes-Côte d’Azur (n = 1,705). Donors provided signed informed consent for the use of their blood samples for nontherapeutic research purposes. This study was approved by the local ethics committee in southern France, Comité de Protection des Personnes Sud Méditerranée I. Blood donors completed a questionnaire that included their year of birth, sex, and detailed information about their lifestyle, environment (home and workplace), and exposure to zoonotic diseases (*22*).

The population in Bolivia comprised 601 voluntary, unpaid, blood donors (*23*) whose serum samples were collected in 2017 in 5 departments: tropical climates of Santa Cruz de la Sierra (n = 165) and Beni (n = 102), Cochabamba (n = 151), and colder subtropical climates (highlands) of Tarija (n = 23) and La Paz (n = 160). This study was approved by the ethics committee of the Medical College of Santa Cruz, and donors provided signed informed consent for research use of their blood samples. The information collected included the year of birth, sex of participants, city of residence, and occupation.

In Laos, collection of blood samples from 657 blood donors was performed in the capital city of Vientiane in 2003, 2004, 2015, and 2018. Donors provided signed informed consent for research use of their blood samples, and the study was approved by the Lao National Health Research Ethics Committee and the Oxford Tropical Research Ethics Committee. Collected information was limited to year of birth and sex of participants.

In Mali, 257 blood samples were collected in 2019 in the villages of Leba, Tliemba, Soloba, Bougoudale, and Komana for a baseline study of health indicators in the villages of the Komana gold mine region (tropical forest area). Participants provided informed consent for research use of their blood samples, and the study was approved by the ethics committee of the National Institute for Public Health Research in Mali. Collected information was limited to the year of birth and sex of participants.

### Seroneutralization Assay

We used the Western Reserve vaccinia virus strain, which is a reference laboratory strain, and the Compiègne strain of cowpox virus that is genetically distant from vaccinia virus. We isolated the Compiègne strain of cowpox virus in 2009 from a human infected by a domestic rat (*24*). We cultured both virus strains on Vero cells in Eagle’s Minimum Essential Medium containing 1% penicillin/streptomycin, 1% glutamine, and 10% fetal calf serum (ThermoFisher Scientific, https://www.thermofisher.com) at 37°C in a 5% CO_2_ incubator. We optimized virus production to obtain low and similar ratios of noninfectious to infectious particles for both strains. For both viruses, we infected Vero cells at 0.01 multiplicity of infection in a 12-well plate for 3 h at 37°C, then washed with Hanks’ Balanced Salt solution. For vaccinia virus, we used clarified supernatant (centrifuged at 700 × *g* for 10 min) collected at 3 days postinfection that titrated at 1.04 × 10^9^ genome copies/mL and 1.67 × 10^6^ 50% tissue culture infectious dose (TCID_50_)/mL (*25*) (ratio of genome copies/TCID_50_ = 624). For cowpox virus, we used clarified supernatant (centrifuged at 700 × *g* for 10 min) collected at 2 days postinfection. We collected cowpox virus 1 day earlier than vaccinia virus because of the large number of noninfectious cowpox virions on day 3 postinfection. The clarified cowpox supernatant titrated at 6.82 × 10^7^ genome copies/mL and 1.08 × 10^5^ TCID_50_/mL (*25*) (ratio = 613). We prepared aliquots in 15 mmol/L HEPES buffer and stored them at –80°C.

We used the same seroneutralization protocol for both viruses. Serum samples were stored at –80°C in specific low binding tubes and thawed before use. We prepared serial dilutions of serum samples in Eagle’s Minimum Essential Medium with 1% penicillin/streptomycin in 96-well plates by using an epMotion 5075 workstation (Eppendorf, https://www.eppendorf.com). We added 50 µL of diluted serum to 50 µL of virus (50 TCID_50_/well) to produce final serum dilutions of 1:20, 1:40, 1:80 and 1:160. We centrifuged the plates at 70 × *g* for 30 s and incubated them for 1 h at 37°C. After neutralization, we added the serum/virus mixtures to 96-well cell culture plates containing confluent Vero cells and 100 µL of culture medium (described previously) and incubated the plates at 37°C in a 5% CO_2_ incubator for 4 d. We included a positive control serum from a donor vaccinated multiple times with the Lister strain of vaccinia virus, which was supplied by the National Reference Centre, France (*26*).

A cytopathic effect appeared on day 3 postinfection for both viruses. We evaluated the plates on day 4 postinfection, and the cytopathic effect was extensive and assisted the analysis. We obtained live cell images by using Cytation (BioTek, https://www.biotek.com) or Incucyte (Sartorius, https://www.sartorius.com) readers. Each image was assigned a result that corresponded to the highest serum dilution that had no cytopathic effect: negative (default value, 1:10) or positive at 1:20, 1:40, 1:80, or 1:160. To assess intraassay reproducibility, we tested 10 replicates of a positive serum sample during the same experiment. To assess interassay reproducibility, we tested 10 replicates of the same serum sample in 5 different experiments (different day and operator). According to criteria classically used for serologic neutralization tests (*27*), we validated the assays by demonstrating that replicate titers were within 3-fold of each other for 80% of tested samples.

### Statistics

We compared the distribution of serologic titers and the proportions of positive and negative serum samples between decades of birth by using Mann-Whitney tests. We compared regions or sex of participants by using Fisher exact test. We calculated geometric means ±SD and created graphs by using GraphPad Prism software (https://www.graphpad.com). We calculated Cohen κ coefficients by using a free online tool (IDoStatistics, https://idostatistics.com/cohen-kappa-free-calculator) to determine correlations between antibodies against vaccinia and cowpox viruses. For samples tested for both viruses, we calculated an orthopoxvirus neutralization titer (ONT) from the geometric mean of vaccinia and cowpox neutralization titers. We performed Mann-Whitney tests for quantitative variables and Fisher exact tests for categorical variables. We compared seroprevalence between each pair of regions by using Fisher exact test without correction for test multiplicity. We identified factors associated with the serologic titer by using univariate analysis, then adjusted for the year of birth by using a parametric model according to the hypothesis of a lognormal distribution of titers and factoring in the interval censoring of serologic titers (*28*). For the study population in France, we analyzed covariates from the questionnaire, including sex, marital status, occupation, level of education, number of persons in the household, household income, general health status, travel outside Europe, housing type, time spent outdoors, air conditioning, mosquito net use, presence of garden/terrace/balcony and swimming pool, dwelling rurality, proximity to shops, presence of a pond or marsh nearby, contact with domestic or farm animals, exposure to mosquitoes or other biting insects and ticks, frequency of bites and protection used, type of water supply, contact with sewage, water consumption, hunting activity, type of meat consumed, and cooking. We used 2-tailed tests for all analyses and defined the significance level as p<0.05.

## Results

### Neutralizing Antibodies against Vaccinia Virus

We determined the male:female ratio, birth decades, and titers for neutralizing antibodies against vaccinia virus for study participants from the different populations in France, Bolivia, Laos, and Mali (Table 1). Seroprevalence was calculated for samples using a threshold titer of >20 (ThT20) or >40 (ThT40).

**Table 1:** Demographic characteristics of participants and results of vaccinia virus neutralization assay in study of orthopoxvirus seroprevalence and infection susceptibility in France, Bolivia, Laos, and Mali*

Characteristics	France	Bolivia	Laos	Mali
Total no. participants	4,876	601	657	255
M/F ratio	1.12	1.37	1.72	1.02
Mean year of birth ±SD	1967 ±14	1989 ±10	1985 ±9	1980 ±14
No. born before 1960	1,560 (32)	3 (0.5)	5 (0.8)	26 (10)
No. born before 1980	3,826 (78)	161 (27)	171 (26)	112 (43)
Antibody titers†				
<20	4,137 (84.84)	598 (99.50)	632 (96.19)	211 (82.74)
20	425 (8.72)	1 (0.17)	22 (3.35)	30 (11.76)
40	217 (4.45)	1 (0.17)	3 (0.46)	12 (4.71)
80	86 (1.76)	1 (0.17)	0 (0)	2 (0.78)
160	11 (0.23)	0 (0)	0 (0)	0 (0)
Titer >40, %	6.44	0.33	0.46	5.49
Titer >20, %	15.16	0.50	3.81	17.25

*Values are no. (%) unless otherwise noted. Percentages were calculated according to the total number of study participants in each population. †No. (%) participants with different levels of neutralizing antibodies against vaccinia virus determined by seroneutralization assay.

### Cross-Immunity between Vaccinia and Cowpox Viruses

A total of 4,448 serum samples from 4 regions of France had sufficient volumes to be tested for both vaccinia and cowpox viruses. We observed that 4,391 (98.8%) of the participants had similar antibody titers for both viruses within +1 dilution. Among 320 participants who had a titer >20 for both viruses, 307 (96%) had concordant titers for both viruses (within +1 dilution) (Table 2). Cohen κ coefficients were 0.43 for the 1:20 titer, 0.64 for the 1:40 titer, 0.48 for the 1:80 titer, and 0.29 for the 1:160 titer. We observed a substantial qualitative agreement between seroneutralization results for vaccinia and cowpox virus at the 1:40 threshold titer, and a concordant titer was found for most samples.

**Table 2:** Cross-reactivity between antibodies against vaccinia and cowpox viruses in serum samples of study participants from France in study of orthopoxvirus seroprevalence and infection susceptibility in France, Bolivia, Laos, and Mali*

Antibody titers, vaccinia virus	Antibody titer, cowpox virus				
	10	20	40	80	160
10	3,443 (77.44)	407 (9.15)	4 (0.09)	2 (0.04)	1 (0.02)
20	234 (5.26)	85 (1.91)	12 (0.27)	5 (0.11)	0
40	7 (0.16)	77 (1.73)	62 (1.39)	12 (0.27)	3 (0.07)
80	0	4 (0.09)	31 (0.70)	21 (0.47)	3 (0.07)
160	0	1 (0.02)	0	2 (0.04)	2 (0.04)

*Values are no. (%). Percentages were calculated according to 4,448 participants in France who had serum samples tested for antibodies against both vaccinia and cowpox viruses by using the seroneutralization assay.

### Epidemiologic Data and Prevalence of Neutralizing Antibodies against Orthopoxviruses in France

Among the 4,448 samples tested for both vaccinia and cowpox virus, the male:female ratio was 1.17 and mean year of birth (+)SD was 1966 (+13) (Table 3). Because of the differences observed between seroprevalence values calculated at ThT20 and ThT40 for vaccinia virus, we determined the ONT and considered samples with an ONT titer >20 to be positive.

**Table 3:** Demographic characteristics of populations in 4 regions of France who had serum samples tested for antibodies against both vaccinia and cowpox viruses in study of orthopoxvirus seroprevalence and infection susceptibility in France, Bolivia, Laos, and Mali*

Characteristics	Corsica	Midi-Pyrénées	PACA	Auvergne-Loire	Total
M/F ratio	1.00	1.16	1.04	1.53	1.17
Mean year of birth ±SD	1966 ±12	1966 ±13	1967 ±13	1966 ±13	1966 ±13
Decade of birth					
1940s	14	185	162	77	438 (9.8)
1950s	38	443	371	213	1,065 (23.9)
1960s	59	457	491	228	1,235 (27.8)
1970s	33	308	359	166	866 (19.5)
1980s	21	244	252	106	623 (14.0)
1990s	3	101	70	47	221 (5.0)
Total	168	1,738	1,705	837	4,448

*Values are no. participants or no. (%) unless otherwise noted. Percentages were calculated according to a total of 4,448 participants in France who had serum samples tested by using the seroneutralization assay. PACA, Provence Alpes Côte-d’Azur.

The mean seroprevalence of orthopoxvirus neutralizing antibodies in France was 8.18%; seroprevalence was >10% in persons born before 1970 and dropped to 5% for those born during the 1970s and to <1% for those born after 1980. The geometric mean ONT for the entire sample population from France was 12.8. We observed limited differences in antibody titers according to age groups, but found a clear overall increase in the percentages of orthopoxvirus-positive persons in relation to age (Figure, panels A, B; Appendix Table 1). 

**Figure:**
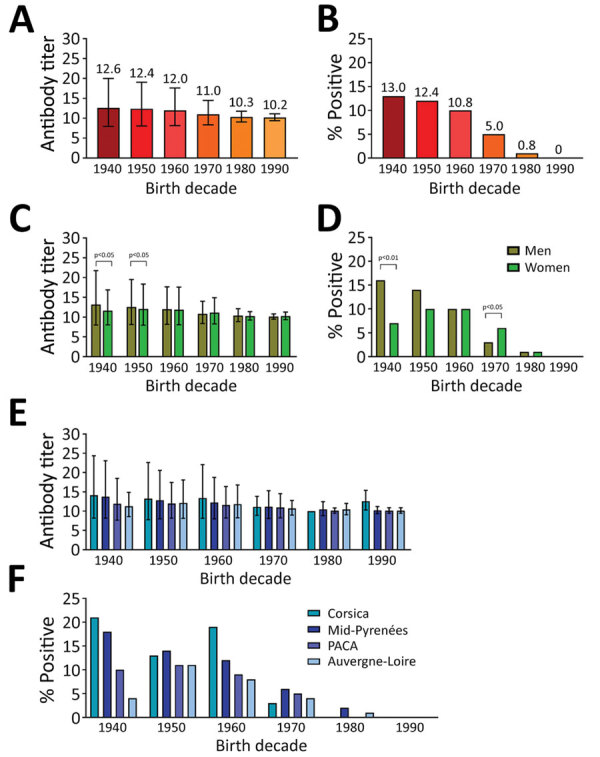
Antibody titers and percentage of population positive for antibodies against orthopoxviruses in France in study of seroprevalence and infection susceptibility in France, Bolivia, Laos, and Mali. Serum samples from 4,448 persons were tested for antibodies against both vaccinia and cowpox virus and an orthopoxvirus neutralization titer (ONT) was determined. A, B) Overall comparison of ONT geometric mean ±SD (A) and percentage of positive participants (ONT>20) (B) according to decade of birth (p values are described in Appendix Table 1). C, D) Comparisons of ONT geometric mean ±SD (C) and percentage of positive persons (ONT>20) (D) between male and female participants according to decade of birth. E, F) Comparisons of ONT geometric mean ±SD (E) and percentage of positive persons (ONT>20) (F) for populations in 4 different regions of France according to their decade of birth (p values are described in Appendix Table 2). Mann-Whitney tests were used to determine geometric means ±SD; Fisher exact tests were used to compare percentage of positive persons. A p value <0.05 was considered significant.

We observed different associations between the sex of study participants and presence of orthopoxvirus neutralizing antibodies in France depending on the decade of birth (Figure, panels C, D). Until the 1960s, orthopoxvirus seroprevalence was higher among men, but it became higher among women in the 1970s. Starting with the 1980s, we observed no difference between sexes. 

The study population in France geographically covered 4 regions. We compared orthopoxvirus neutralizing antibody titers and percentages of seropositive participants in the different regions for each decade (Figure, panels E, F; Appendix Table 2). We observed some differences in antibody titers and percentages of seropositive persons between the regions. Seroprevalence was higher in Corsica and the Midi-Pyrénées regions than in the Provence-Alpes-Côte d’Azur and Auvergne-Loire regions.

During the collection of samples, participants in France filled in a detailed questionnaire concerning lifestyle, eating habits, rurality index, contact with livestock or wild animals, and other personal information. We estimated the association between each of these parameters and ONT. We evaluated 60 parameters and determined none of the parameters influenced seroprevalence after adjusting for age. The only parameter directly associated with the titer of orthopoxvirus neutralizing antibodies was age.

## Discussion

After the end of smallpox vaccinations in the 1980s, prevalence of antibodies against orthopoxviruses was expected to decline worldwide; seroprevalence would decrease in the youngest (and never vaccinated) age groups. Furthermore, circulation of orthopoxviruses other than smallpox was expected to contribute to immunity against smallpox by natural infection in some persons. Similarly, whereas smallpox vaccination can produce long-lasting immunity, immunosenescence in older vaccinated populations was expected to contribute to a decrease in seroprevalence in persons vaccinated in childhood. Consequently, prevalence of antibodies against orthopoxviruses is difficult to anticipate precisely in a given population because parameters that can modulate seroprevalence are numerous, including intensity of past vaccination campaigns, number of doses received, possible exposure to orthopoxvirus infections, and immunosenescence. Our study provides concrete information on seroprevalence of antibodies against orthopoxviruses and compares several populations on different continents.

Overall, our results are broadly consistent with the expectations described above. Seroprevalence of orthopoxvirus-specific antibodies was higher in countries that routinely vaccinated their population and in study participants born before the cessation of smallpox vaccinations. However, several points should be discussed.

Results from Bolivia and Laos are consistent with the previous World Health Organization containment strategy used when vaccination of the general population was not feasible. Orthopoxvirus seroprevalence was remarkably low in Bolivia (<0.5%, regardless of the threshold titer used) in those participants born before and after 1980. This result likely reflects both low vaccination coverage and lack of exposure to natural orthopoxvirus infections.

In Laos, overall seroprevalence using ThT40 was very low (<1%) but similar to ThT20 (4%). Prevalence values for persons born before 1980 were 1.8% (ThT40) and 7.0% (ThT20) compared with 0% (ThT40) and 2.7% (ThT20) for those born after 1980 (Appendix Table 3). Therefore, neutralizing antibodies against orthopoxviruses are found predominantly (but not exclusively) in persons born before 1980 and might be related to smallpox vaccination, although low-level exposure to other orthopoxviruses cannot be excluded.

In Mali, overall seroprevalence of orthopoxvirus antibodies using ThT40 was ≈5% and increased to ≈17% using ThT20. Seroprevalence values for persons born before 1980 were 10.7% (ThT40) and 27.7% (ThT20) compared with 1.4% (ThT40) and 9.0% (ThT20) for those born after 1980 (Appendix Table 3). Thus, seroprevalence was higher in older participants, but a substantial number of participants born after 1980 had neutralizing antibodies against vaccinia virus. Health authorities confirmed that no smallpox vaccination campaign existed in the study area after 1980. The distribution of seroprevalence in Mali for different age groups using ThT20 (Appendix Figure) suggests that exposure to natural orthopoxvirus infections accounts for part of the observed immunity. Samples were collected from persons in villages located in a forested area (southern Mali), and circulation of monkeypox virus in humans, monkeys, and rodents has been reported in central and western Africa for several decades, primarily at the edge of forests (*3*). However, vaccination might also explain the high prevalence of orthopoxvirus antibodies in the oldest age groups.

In France, we had the opportunity to perform testing for both vaccinia and cowpox viruses in a large portion of our study population to improve the specificity of the neutralization assay. Our results for both viruses were similar and had a substantial qualitative agreement and concordant titers consistent with previously documented cross-immunity between orthopoxviruses (*29*). We found an orthopoxvirus antibody seroprevalence of 8.18%, which was mainly related to age and, thus, to smallpox vaccination coverage, and a sharp decline in prevalence beginning in the 1970s. We observed differences between sexes; antibodies were higher in men, especially those who were born before 1960. We suspect that the medical rigor resulting from military service for men in France might have contributed to higher seroprevalence in the older age groups.

In our study, we observed that some participants born in the early 1940s still had high neutralization titers for orthopoxviruses. This result is consistent with previous reports that smallpox vaccination generates long-term splenic memory lymphocytes that can lead to the production of antibodies against the vaccine >80 years after vaccination (*30*–*39*). However, we also show a low seroprevalence among participants born before 1960 (≈12.5%). Methodologically similar studies have also reported that age is the main factor affecting prevalence and antibody titer. The overall rate and decrease in prevalence is highly variable according to country (and likely vaccination policy) and serologic methods used for testing. In Japan (*40*), the reported prevalence of neutralizing antibodies was high (>90%) for persons born before 1968 and then decreased sharply in the 1970s after the cessation of smallpox vaccination in 1976. A different pattern was observed in Australia (*41*); prevalence of antibodies was 48% among persons born before 1950 and progressively decreased by half each decade for persons born after 1950. The prevalence pattern in France is similar to that in Australia; initial vaccination coverage was estimated at 90% by Santé Publique France among persons born before 1966 (*20*), but the current seroprevalence was reported to be lower than expected (*36*). The remnants of humoral smallpox immunity appear to be conditioned by multiple factors, such as vaccine policy and population immunity. For previously vaccinated persons in whom no neutralizing antibodies have been found, the extent to which they retain functional immunity against orthopoxviruses remains unknown.

Our results also show that compliance with smallpox vaccination or booster shots in France declined well before the end of compulsory vaccination, and territorial disparities might exist. Smallpox disappeared from Europe after World War I, and the epidemics generated by imported cases in the 1950s (*42*,*43*) consistently suggest that vaccination coverage had already begun to decline, possibly driven by adverse effects of the vaccine.

Of note, the ThT20 prevalence values in persons born after 1980 were low (1.74% in Midi-Pyrénées, 0.65% in Auvergne-Loire, and 0% in Provence-Alpes-Côte d’Azur), suggesting the absence of natural orthopoxvirus infections. Further investigations with a larger number of study participants are needed to clarify the proportion of persons in Corsica born after 1980 with antibodies to vaccinia virus. No environmental factors were associated with antibody seroprevalence in the study population in France, despite a large database on living conditions.

The first limitation of our study is that the tested cohorts might not be representative of the general population. Differences existed in the recruitment of participants from the different international populations, and a limited number of older persons were tested. Second, individual vaccine data and collection of metadata were absent or weak. Finally, a strict internationally validated threshold value for neutralization tests was absent.

In conclusion, our study suggests that, overall, the different populations that we tested in Europe, Africa, Asia, and South America are markedly susceptible to orthopoxvirus infections. Even in Africa, where substantial evidence of natural circulation of orthopoxviruses exists, population immunity is modest. Levels of protection against orthopoxvirus infection are lowest in persons born after 1980 because smallpox vaccinations were discontinued. In practical terms, population immunity that might provide a barrier to the spread of orthopoxviruses does not appear to exist. Our study indicates that cessation of smallpox vaccinations might precipitate the emergence of orthopoxvirus infections, such as the currently observed spread of monkeypox in Europe.

Appendix Additional information for orthopoxvirus seroprevalence and infection susceptibility in France, Bolivia, Laos, and Mali.

## References

[1] Beer EM, Rao VB. A systematic review of the epidemiology of human monkeypox outbreaks and implications for outbreak strategy. PLoS Negl Trop Dis. 2019;13:e0007791. 10.1371/journal.pntd.0007791 31618206 PMC6816577

[2] Doshi RH, Guagliardo SAJ, Doty JB, Babeaux AD, Matheny A, Burgado J, et al. Epidemiologic and ecologic investigations of monkeypox, Likouala Department, Republic of the Congo, 2017. Emerg Infect Dis. 2019;25:281–9. 10.3201/eid2502.181222 30666937 PMC6346463

[3] Durski KN, McCollum AM, Nakazawa Y, Petersen BW, Reynolds MG, Briand S, et al. Emergence of Monkeypox - West and Central Africa, 1970-2017. MMWR Morb Mortal Wkly Rep. 2018;67:306–10. 10.15585/mmwr.mm6710a5 29543790 PMC5857192

[4] Centers for Disease Control and Prevention (CDC). Update: multistate outbreak of monkeypox—Illinois, Indiana, Kansas, Missouri, Ohio, and Wisconsin, 2003. MMWR Morb Mortal Wkly Rep. 2003;52:589–90. 12836628

[5] Vaughan A, Aarons E, Astbury J, Balasegaram S, Beadsworth M, Beck CR, et al. Two cases of monkeypox imported to the United Kingdom, September 2018. Euro Surveill. 2018;23:1800509. 10.2807/1560-7917.ES.2018.23.38.1800509 30255836 PMC6157091

[6] Venkatesan G, Balamurugan V, Prabhu M, Yogisharadhya R, Bora DP, Gandhale PN, et al. Emerging and re-emerging zoonotic buffalopox infection: a severe outbreak in Kolhapur (Maharashtra), India. Vet Ital. 2010;46:439–48. 21120799

[7] Kolhapure RM, Deolankar RP, Tupe CD, Raut CG, Basu A, Dama BM, et al. Investigation of buffalopox outbreaks in Maharashtra State during 1992-1996. Indian J Med Res. 1997;106:441–6. 9415737

[8] Prabhu M, Yogisharadhya R, Pavulraj S, Suresh C, Sathish G, Singh RK. Camelpox and buffalopox: two emerging and re-emerging orthopox viral diseases of India. Adv Anim Vet Sci. 2015;3:527–41. 10.14737/journal.aavs/2015/3.10.527.541

[9] Balamurugan V, Venkatesan G, Bhanuprakash V, Singh RK. Camelpox, an emerging orthopox viral disease. Indian J Virol. 2013;24:295–305. 10.1007/s13337-013-0145-0 24426291 PMC3832703

[10] Bera BC, Shanmugasundaram K, Barua S, Venkatesan G, Virmani N, Riyesh T, et al. Zoonotic cases of camelpox infection in India. Vet Microbiol. 2011;152:29–38. 10.1016/j.vetmic.2011.04.010 21571451

[11] Duraffour S, Mertens B, Meyer H, van den Oord JJ, Mitera T, Matthys P, et al. Emergence of cowpox: study of the virulence of clinical strains and evaluation of antivirals. PLoS One. 2013;8:e55808. 10.1371/journal.pone.0055808 23457480 PMC3574090

[12] Kurth A, Wibbelt G, Gerber HP, Petschaelis A, Pauli G, Nitsche A. Rat-to-elephant-to-human transmission of cowpox virus. Emerg Infect Dis. 2008;14:670–1. 10.3201/eid1404.070817 18394293 PMC2570944

[13] Wolfs TFW, Wagenaar JA, Niesters HGM, Osterhaus ADME. Rat-to-human transmission of Cowpox infection. Emerg Infect Dis. 2002;8:1495–6. 10.3201/eid0812.020089 12498670 PMC2738512

[14] Keesing F, Belden LK, Daszak P, Dobson A, Harvell CD, Holt RD, et al. Impacts of biodiversity on the emergence and transmission of infectious diseases. Nature. 2010;468:647–52. 10.1038/nature09575 21124449 PMC7094913

[15] Keesing F, Ostfeld RS. Impacts of biodiversity and biodiversity loss on zoonotic diseases. Proc Natl Acad Sci U S A. 2021;118:e2023540118. 10.1073/pnas.2023540118 33820825 PMC8092607

[16] Schmeller DS, Courchamp F, Killeen G. Biodiversity loss, emerging pathogens and human health risks. Biodivers Conserv. 2020;29:3095–102. 10.1007/s10531-020-02021-6 32836920 PMC7423499

[17] Berche P. The threat of smallpox and bioterrorism. Trends Microbiol. 2001;9:15–8. 10.1016/S0966-842X(00)01855-2 11166237

[18] Cohen J. Bioterrorism. Smallpox vaccinations: how much protection remains? Science. 2001;294:985. 10.1126/science.294.5544.985 11691969

[19] Lane HC, Montagne JL, Fauci AS. Bioterrorism: a clear and present danger. Nat Med. 2001;7:1271–3. 10.1038/nm1201-1271 11726956

[20] Lévy-Bruhl D, Guérin N; Members of the Eurosurveillance editorial board. The use of smallpox virus as a biological weapon: the vaccination situation in France. Euro Surveill. 2001;6:171–8. 10.2807/esm.06.11.00385-en 11891388

[21] Fenner F, Henderson DA, Arita I, Jezek Z, Ladnyi ID. Smallpox and its eradication. Geneva: World Health Organization; 1988.

[22] Poinsignon A, Boulanger D, Binetruy F, Elguero E, Darriet F, Gallian P, et al. Risk factors of exposure to *Aedes albopictus* bites in mainland France using an immunological biomarker. Epidemiol Infect. 2019;147:e238. 10.1017/S0950268819001286 31364567 PMC6625181

[23] Saba Villarroel PM, Nurtop E, Pastorino B, Roca Y, Drexler JF, Gallian P, et al. Zika virus epidemiology in Bolivia: A seroprevalence study in volunteer blood donors. PLoS Negl Trop Dis. 2018;12:e0006239. 10.1371/journal.pntd.0006239 29513667 PMC5858838

[24] Ninove L, Domart Y, Vervel C, Voinot C, Salez N, Raoult D, et al. Cowpox virus transmission from pet rats to humans, France. Emerg Infect Dis. 2009;15:781–4. 10.3201/eid1505.090235 19402968 PMC2686997

[25] Reed LJ, Muench H. A simple method of estimating fifty percent endpoints. Am J Epidemiol. 1938;27:493–7. 10.1093/oxfordjournals.aje.a118408

[26] Leparc-Goffart I, Poirier B, Garin D, Tissier MH, Fuchs F, Crance JM. Standardization of a neutralizing anti-vaccinia antibodies titration method: an essential step for titration of vaccinia immunoglobulins and smallpox vaccines evaluation. J Clin Virol. 2005;32:47–52. 10.1016/j.jcv.2004.07.005 15572006

[27] Timiryasova TM, Bonaparte MI, Luo P, Zedar R, Hu BT, Hildreth SW. Optimization and validation of a plaque reduction neutralization test for the detection of neutralizing antibodies to four serotypes of dengue virus used in support of dengue vaccine development. Am J Trop Med Hyg. 2013;88:962–70. 10.4269/ajtmh.12-0461 23458954 PMC3752766

[28] Lapidus N, de Lamballerie X, Salez N, Setbon M, Delabre RM, Ferrari P, et al. Factors associated with post-seasonal serological titer and risk factors for infection with the pandemic A/H1N1 virus in the French general population. PLoS One. 2013;8:e60127. 10.1371/journal.pone.0060127 23613718 PMC3629047

[29] Edghill-Smith Y, Golding H, Manischewitz J, King LR, Scott D, Bray M, et al. Smallpox vaccine-induced antibodies are necessary and sufficient for protection against monkeypox virus. Nat Med. 2005;11:740–7. 10.1038/nm1261 15951823

[30] el-Ad B, Roth Y, Winder A, Tochner Z, Lublin-Tennenbaum T, Katz E, et al. The persistence of neutralizing antibodies after revaccination against smallpox. J Infect Dis. 1990;161:446–8. 10.1093/infdis/161.3.446 2155973

[31] Demkowicz WE Jr, Littaua RA, Wang J, Ennis FA. Human cytotoxic T-cell memory: long-lived responses to vaccinia virus. J Virol. 1996;70:2627–31. 10.1128/jvi.70.4.2627-2631.1996 8642697 PMC190113

[32] Crotty S, Felgner P, Davies H, Glidewell J, Villarreal L, Ahmed R. Cutting edge: long-term B cell memory in humans after smallpox vaccination. J Immunol. 2003;171:4969–73. 10.4049/jimmunol.171.10.4969 14607890

[33] Pütz MM, Alberini I, Midgley CM, Manini I, Montomoli E, Smith GL. Prevalence of antibodies to Vaccinia virus after smallpox vaccination in Italy. J Gen Virol. 2005;86:2955–60. 10.1099/vir.0.81265-0 16227216

[34] Hammarlund E, Lewis MW, Hansen SG, Strelow LI, Nelson JA, Sexton GJ, et al. Duration of antiviral immunity after smallpox vaccination. Nat Med. 2003;9:1131–7. 10.1038/nm917 12925846

[35] Combadiere B, Boissonnas A, Carcelain G, Lefranc E, Samri A, Bricaire F, et al. Distinct time effects of vaccination on long-term proliferative and IFN-γ-producing T cell memory to smallpox in humans. J Exp Med. 2004;199:1585–93. 10.1084/jem.20032083 15184506 PMC2211784

[36] Taub DD, Ershler WB, Janowski M, Artz A, Key ML, McKelvey J, et al. Immunity from smallpox vaccine persists for decades: a longitudinal study. Am J Med. 2008;121:1058–64. 10.1016/j.amjmed.2008.08.019 19028201 PMC2610468

[37] Sarkar JK, Mitra AC, Mukherjee MK. The minimum protective level of antibodies in smallpox. Bull World Health Organ. 1975;52:307–11. 1084801 PMC2366379

[38] Amanna IJ, Carlson NE, Slifka MK. Duration of humoral immunity to common viral and vaccine antigens. N Engl J Med. 2007;357:1903–15. 10.1056/NEJMoa066092 17989383

[39] Mamani-Matsuda M, Cosma A, Weller S, Faili A, Staib C, Garçon L, et al. The human spleen is a major reservoir for long-lived vaccinia virus-specific memory B cells. Blood. 2008;111:4653–9. 10.1182/blood-2007-11-123844 18316630

[40] Hatakeyama S, Moriya K, Saijo M, Morisawa Y, Kurane I, Koike K, et al. Persisting humoral antiviral immunity within the Japanese population after the discontinuation in 1976 of routine smallpox vaccinations. Clin Diagn Lab Immunol. 2005;12:520–4. 15817760 10.1128/CDLI.12.4.520-524.2005 PMC1074390

[41] Costantino V, Trent MJ, Sullivan JS, Kunasekaran MP, Gray R, MacIntyre R. Serological immunity to smallpox in New South Wales, Australia. Viruses. 2020;12:554. 10.3390/v12050554 32443405 PMC7291091

[42] Biraben JN. La diffusion de la vaccination en France au 19e siècle. [in French]. Ann Bretagne Payes Ouest. 1979;86:265–76. 10.3406/abpo.1979.2981 11631442

[43] Le Boukdelles B. The smallpox epidemic of 1955 in France [in French]. Bull Acad Natl Med. 1955;139:417–20. 13284423

