# Supplementary material for: Orthopoxvirus Seroprevalence and Infection Susceptibility in France, Bolivia, Laos, and Mali
Source: Emerg Infect Dis. 2022 Dec;28(12):2463–71. doi: 10.3201/eid2812.221136 (PMC9707606; doi:10.3201/eid2812.221136)
Supplement: Appendix — Additional information for orthopoxvirus seroprevalence and infection susceptibility in France, Bolivia, Laos, and Mali. [file 22-1136-Techapp-s1.pdf]

Article DOI: https://doi.org/10.3201/eid2812.221136

Orthopoxvirus Seroprevalence and Infection Susceptibility in France, Bolivia, Laos, and Mali

Appendix

Appendix Table 1. The p values associated with Figure, panels A, B (main text), showing comparisons of antibody titers and % orthopoxvirus-positive populations in France according to decade of birth in study of orthopoxvirus seroprevalence and infection susceptibility in France, Bolivia, Laos, and Mali*

Birth decade	Antibody titer p values†					% Positive p values†				
	Birth decade					Birth decade				
	1940s	1950s	1960s	1970s	1980s	1940s	1950s	1960s	1970s	1980s
1950s	0.856	NA	NA	NA	NA	0.733	NA	NA	NA	NA
1960s	0.082	0.039	NA	NA	NA	0.131	0,113	NA	NA	NA
1970s	<0.0001	<0.0001	<0.0001	NA	NA	<0.0001	<0.0001	<0.0001	NA	NA
1980s	<0.0001	<0.0001	<0.0001	<0.0001	NA	<0.0001	<0.0001	<0.0001	<0.0001	NA
1990s	<0.0001	<0.0001	<0.0001	0.0002	0.535	<0.0001	<0.0001	<0.0001	<0.0001	0.334

*Comparisons between different birth decades were performed using the Mann-Whitney test. Comparisons were performed for study participants in France who had serum samples tested for both vaccinia and cowpox viruses. NA, not applicable.
†p<0.05 was considered significant.

Appendix Table 2. The p values associated with Figure, panels E, F (main text), showing comparisons of antibody titers and % orthopoxvirus-positive persons between 4 regions of France according to decades of birth in study of orthopoxvirus seroprevalence and infection susceptibility in France, Bolivia, Laos, and Mali*

	Antibody titer p values†			% Positive p values†		
Birth decade	Corsica	Midi-Pyrénées	PACA	Corsica	Midi-Pyrénées	PACA
1940s						
Midi- Pyrénées	0.851	NA	NA	0.727	NA	NA
PACA	0.068	0.0003	NA	0.201	0.048	NA
Auvergne-Loire	0.109	0.005	0.896	0.045	0.002	0.131
1950s						
Midi- Pyrénées	0.616	NA	NA	>0,999	NA	NA
PACA	0.124	0.021	NA	0.599	0.206	NA
Auvergne-Loire	0.235	0.165	0.581	0.588	0.266	>0.999
1960s						
Midi- Pyrénées	0.162	NA	NA	0.145	NA	NA
PACA	0.015	0.047	NA	0.035	0.166	NA
Auvergne-Loire	0.088	0.526	0.311	0.026	0.145	0.671
1970s						
Midi- Pyrénées	0.323	NA	NA	>0.999	NA	NA
PACA	0.198	0.457	NA	>0.999	0.732	NA
Auvergne-Loire	0.274	0.707	0.813	>0.999	0.381	0.654
1980s						
Midi- Pyrénées	0.155	NA	NA	>0.999	NA	NA
PACA	0.617	0.004	NA	>0.999	>0.999	NA
Auvergne-Loire	0.209	0.924	0.018	>0.999	>0.999	0.296
1990s						
Midi- Pyrénées	0.019	NA	NA	>0.999	NA	NA
PACA	0.011	0.529	NA	>0.999	>0.999	NA
Auvergne-Loire	0.014	0.719	>0.999	>0.999	>0.999	>0.999

*Comparisons between different birth decades were performed using Mann-Whitney tests. NA, not applicable; PACA, Provence Alpes Côte-d'Azur.
†p<0.05 was considered significant.

Appendix Table 3. Prevalence of antibodies against orthopoxvirus in persons from Mali and Laos born before or after 1980 using different titer thresholds in study of orthopoxvirus seroprevalence and infection susceptibility in France, Bolivia, Laos, and Mali*

Population	% Born before 1980	% Born after 1980
Mali		
ThT20	27.7	9.0
ThT40	10.7	1.4
Laos		
ThT20	7.0	2.7
ThT40	1.8	0
Bolivia		
NA	NA	NA

*NA, not applicable; ThT20, threshold titer 1:20; ThT40, threshold titer 1:40.

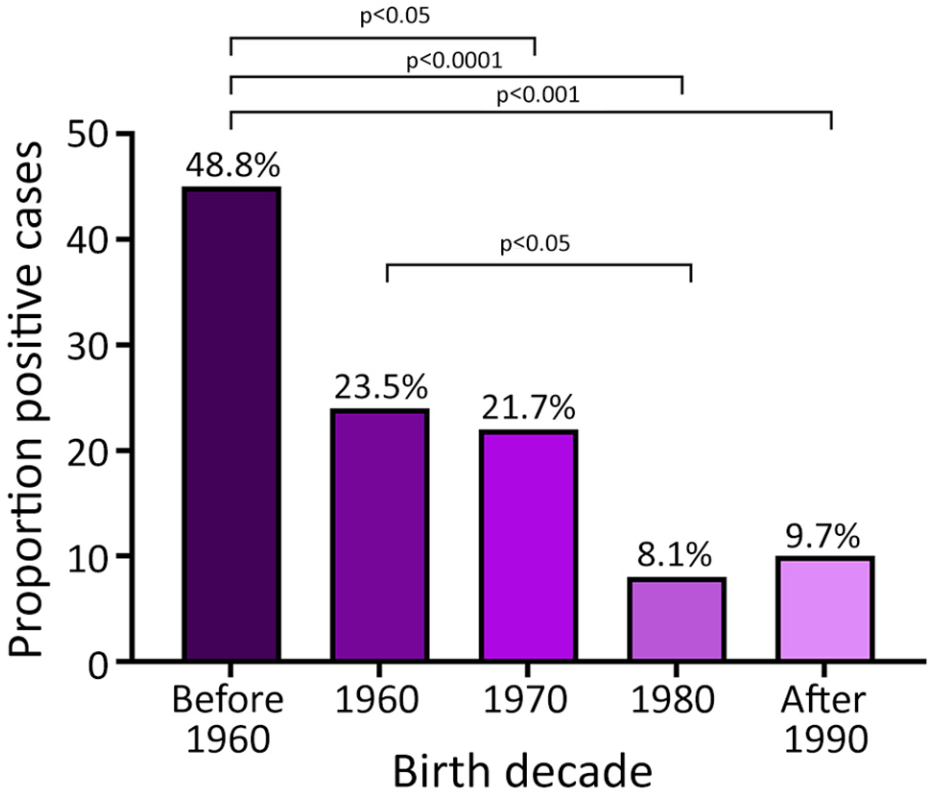

Appendix Figure. Percentage of participants in Mali who had serum samples with antibodies against orthopoxvirus according to the decade of birth in study of seroprevalence and infection susceptibility in France, Bolivia, Laos, and Mali. The threshold titer to determine positivity was 1:20. The numbers of participants for each birth decade are: born before 1960, n = 26; born in 1960s, n = 34; born in 1970s, n = 46; born in 1980s, n = 74; born after 1990, n = 72. Fisher exact tests were used to determine differences; $p<0.05$ was considered significant.
